# Functional markers for gene mapping and genetic diversity studies in sugarcane

**DOI:** 10.1186/1756-0500-4-264

**Published:** 2011-07-28

**Authors:** Thiago G Marconi, Estela A Costa, Hercília RCAN Miranda, Melina C Mancini, Cláudio B Cardoso-Silva, Karine M Oliveira, Luciana R Pinto, Marcelo Mollinari, Antônio AF Garcia, Anete P Souza

**Affiliations:** 1 Centro de Biologia Molecular e Engenharia Genética (CBMEG) - Universidade Estadual de Campinas (UNICAMP), Cidade Universitária Zeferino Vaz, CP 6010, CEP 13083-970, Campinas, SP, Brazil; 2 Centro Avançado da Pesquisa Tecnológica do Agronegócio de Cana - IAC/Apta, Anel Viário Contorno Sul, Km 321, CP 206, CEP 14.001-970, Ribeirão Preto, SP, Brazil; 3 Departamento de Genética, Escola Superior de Agricultura Luiz de Queiroz (ESALQ), Universidade de São Paulo (USP), CP 83, CEP 13400-970, Piracicaba, SP, Brazil; 4 Departamento de Biologia Vegetal, Instituto de Biologia, Universidade Estadual de Campinas (UNICAMP), Cidade Universitária Zeferino Vaz, CP 6109, CEP 13083-970, Campinas, SP, Brazil; 5 Centro de Tecnologia Canavieira - CTC, Caixa Postal 162, 13400-970, Piracicaba, SP, Brazil

## Abstract

**Background:**

The database of sugarcane expressed sequence tags (EST) offers a great opportunity for developing molecular markers that are directly associated with important agronomic traits. The development of new EST-SSR markers represents an important tool for genetic analysis. In sugarcane breeding programs, functional markers can be used to accelerate the process and select important agronomic traits, especially in the mapping of quantitative traits loci (QTL) and plant resistant pathogens or qualitative resistance loci (QRL). The aim of this work was to develop new simple sequence repeat (SSR) markers in sugarcane using the sugarcane expressed sequence tag (SUCEST database).

**Findings:**

A total of 365 EST-SSR molecular markers with trinucleotide motifs were developed and evaluated in a collection of 18 genotypes of sugarcane (15 varieties and 3 species). In total, 287 of the EST-SSRs markers amplified fragments of the expected size and were polymorphic in the analyzed sugarcane varieties. The number of alleles ranged from 2-18, with an average of 6 alleles per locus, while polymorphism information content values ranged from 0.21-0.92, with an average of 0.69. The discrimination power was high for the majority of the EST-SSRs, with an average value of 0.80. Among the markers characterized in this study some have particular interest, those that are related to bacterial defense responses, generation of precursor metabolites and energy and those involved in carbohydrate metabolic process.

**Conclusions:**

These EST-SSR markers presented in this work can be efficiently used for genetic mapping studies of segregating sugarcane populations. The high Polymorphism Information Content (PIC) and Discriminant Power (DP) presented facilitate the QTL identification and marker-assisted selection due the association with functional regions of the genome became an important tool for the sugarcane breeding program.

## Background

Sugarcane (*Saccharum *spp.) is a member of a polyploid complex belonging to the Andropogoneae tribe in the Poaceae family. This crop is economically important because it is the main source of both sugar and alcohol, and it accounts for two thirds of the world's sugar production [1]. In Brazil, the production about 625 million tons of sugarcane is estimated for the 2010/2011 harvest, surpassing the production of the last harvest [2].

Modern sugarcane cultivars are highly polyploid and often aneuploid, with chromosome numbers ranging from 100 to 130 [3]. These modern cultivars are interspecific hybrids derived essentially from crosses between *S. officinarum *(2n = 80, x = 10), a species that has stalks with high sugar content, and *S. spontaneum *(2n = 40-128, x = 8), a wild and vigorous species that is resistant to several sugarcane diseases. Because of sugarcane's interspecific origins and its high polyploid chromosome number, crosses between different sugarcane varieties produce aneuploid progeny [4,5]. These characteristics make the time required for developing new varieties as high as 12 to 15 years [6], and they represent some of the main drawbacks in sugarcane breeding programs.

Molecular markers associated with important agronomic traits can be used to help select varieties at early stages of breeding programs, known as Marker-Assisted Selection (MAS) and to choose the best parents in a cross. Therefore, they could facilitate a significant reduction in the time and cost involved to develop new varieties, and they should help to bypass barriers in sugarcane breeding.

Simple sequence repeats (SSR) or microsatellites are short fragments of DNA that consist of small motifs of one to six tandem repeat base pairs that are flanked by well-conserved sequences, and they allow for the design of specific primers [7-9]. The SSRs developed from expressed sequence tags (ESTs) are derived from expressed genes. The advantage of using ESTs is that they can facilitate the mapping of genes with known functions pathways [10]. They are also located in the transcribed portion of the genome [11], which allows for a direct association between genes and important agronomic traits. ESTs have been developed for a large number of plant species, including cotton [12], wheat [13], barley [14], and the rubber tree [15].

To identify available ESTs, our group used the Brazilian EST database (Sugarcane EST Project- SUCEST), which is the largest database of sugarcane ESTs; it contains 237,954 ESTs grouped in 43,000 clusters [16]. Most of these clusters represent genes associated with important metabolic processes such as photosynthesis, carbohydrate metabolism, sugar transport, amino acid metabolism, and biotic and abiotic stress response mechanisms.

A preliminary analysis involving the development of functional SSRs markers from the SUCEST database was reported by Da Silva [17], who identified 20 EST-SSRs revealing this database as a good source for the development of molecular markers.

In total, our group has developed 837 sugarcane EST-SSRs. Initially were reported the development and characterization of 30 EST-SSRs [18]. Later, developed 100 more EST-SSRs and compared them with 50 other SSRs derived from genomic libraries (gSSR) [19]. After, developed 149 additional EST-SSRs [20], and more recently, developed 193 EST-SSRs [21]. In the present article, we develop and characterize 365 novel EST-SSRs.

The aim of the present work was to characterize 365 SSR sugarcane markers identified in the SUCEST database. Appropriate primers and PCR amplification conditions were developed for each SSR, and their polymorphism information content (PIC) and discrimination power (DP) were determined. To validate the newly developed EST-SSRs, the genetic diversity among 18 sugarcane genotypes (15 varieties and 3 species) was evaluated and the results were compared with previously reported data that utilized AFLPs and pedigrees [22].

## Methods

### Plant Material and DNA Extraction

A total of 13 commercial sugarcane varieties (*CB 36-24, SP 79-1011, CB 40-77, IAC 51-205, RB 73-9359, SP 70-1284, IAC 64-257, SP 71-1406, SP 80-3280, RB 85-5035, SP 79-6134, SP 79-2312*, and *RB 85-5536*) and two parental varieties (*SP 80-180 *and *SP 80-4966*) from a genetic mapping program [20,23] at Centro de Tecnologia Canavieira (CTC - Piracicaba, SP, Brazil) and one clone from each of three *Saccharum *species (*IJ76-314, S. officinarum*; *Gandacheni, S. barberi*; and *Maneria, S. sinense*) (Table 1) were used to validate the developed EST-derived markers and to screen for polymorphic SSRs. The studied germplasm was chosen according to a previous study on sugarcane genetic diversity that utilized AFLP markers and pedigree information [22]. Genomic DNA was extracted from 300 mg of lyophilized young leaf tissue using the method described by Hoisington et al. [24] with minor modifications.

**Table 1:** Sugarcane genotypes used for marker validation with pedigree relationships covering two generations and their origins

Genotypes	Pedigree	Origin
CB36-24	[POJ2364 × EK28] POJ2878 × ?	Campos, Brazil
CB40-77	[POJ2364 × EK28] POJ2878 × CO290 [CO221 × D74]	Campos, Brazil
SP79-1011	[CO419 × CO419] NA5679 × CO775 [POJ2878 × CO371]	São Paulo, Brazil
SP70-1284	[POJ2878 × ?] B4176 × ?	São Paulo, Brazil
SP71-1406	[CO419 × CO419] NA5679 × ?	São Paulo, Brazil
SP80-3280	[CP5530 × CP5376] SP71-1088 × H575028 [H49134 × ?]	São Paulo, Brazil
SP79-6134	[H53263 × H507209] H634644 × ?	São Paulo, Brazil
SP79-2312	[SP71-6106 × ?] CP5659 × ?	São Paulo, Brazil
SP80-4966	[NA5679 × ?] SP711406 × ?	São Paulo, Brazil
SP80-180	[B3337 × ?] B3337 × ?	São Paulo, Brazil
IAC51-205	[POJ2364 × EK28] POJ2878 × ?	Campinas, Brazil
IAC64-257	[POJ2878 × CO290] CO419 × IAC49-131 [CP27108 × ?]	Campinas, Brazil
RB739359	[CO419 × MZ336] IANE5534 × ?	Republic of Brazil
RB855035	[CP521 × CP48103] L6014 × SP701284 [CB4176 × ?]	Republic of Brazil
RB855536	[IAC48-65 × ?] SP70-1143 × RB72454 [CP5376 × ?]	Republic of Brazil
Gandacheni (*S. barberi*)	*S. barberi *× ?	Saretha, Índia
IJ76-314 (*S. officinarum*)	*S. officinarum *× ?	Iryan, Java
Maneria (*S. sinense*)	*S. sinense *× ?	Pansahi, China

### Development of SSR markers

SSR markers were designed from 2005 sugarcane consensus sequences derived from the SUCEST database (The Sugarcane EST project), as described in detail by Pinto et al. [18]. A set of 365 locus-specific primers flanking SSRs with trinucleotide motifs was designed using the Primer Select DNAStar^® ^package software. The primers were synthesized by Integrated DNA Technologies (IDT) (São Paulo, SP, Brazil). The criteria used to design the primers for SSR flanking regions were the same as those described by Cordeiro et al. [25].

To evaluate the importance of these EST-SSR markers, they were compared to those in the GenBank database using BLASTX directly from NCBI. The biological function was identified using the Gene Ontology database.

### PCR amplification and EST-SSR visualization

PCR amplifications were performed in 20 μl reaction containing 10× PCR buffer (10 mM Tris-HCl, 50 mM KCl), 2.0 mM MgCl_2_, 100 μM of each dNTP, 0.2 μM of each forward and reverse primer, 0.5 U Taq DNA polymerase (Invitrogen, SP, Brazil), and 40 ng of template DNA. After an initial denaturation at 94°C for 3 min, DNA fragments were PCR-amplified for 31 cycles of 1 min at 94°C, 1 min at the annealing temperature specific to each primer, and 1 min at 72°C; the final extension time was for 2 min at 72°C. The annealing temperature for each primer pair was determined using a temperature gradient ranging from 50 to 65°C on a PTC-200 thermocycler (MJ Research). The amplification products were separated by electrophoresis on 6% denaturing polyacrylamide gels in 1× TBE buffer. To perform the electrophoresis, PCR reaction mixtures were mixed with equal volumes of loading buffer (formamide containing 0.8 mM EDTA and traces of bromophenol blue and xylene cyanol) and denatured at 90°C for 3 min. Samples were then loaded on a pre-heated S2001 Model (Life Technologies). A 10 bp ladder was used (Invitrogen, SP, Brazil) as a size standard. The DNA fragments were visualized by silver staining according to Creste et al. [26].

### Statistical analysis

#### Polymorphism analysis

All segregating unique bands were treated as dominant markers because sugarcane is a hybrid species that incorporates multiple chromosomes sets that can pair and recombine freely, that is, with little or no preferential pairing. The data were scored based on the presence (1) or absence (0) of a band for each of the 18 sugarcane genotypes. Both PIC and DP were obtained for a polymorphism analysis of each locus. PIC values were calculated based on the following formula:

where *p*_*i *_is the frequency of the *i*^th ^allele and the summation extends over *n *alleles [27]. DP values for the *k*^th ^primer were calculated based on the following formula:

where *N *is the number of individuals and *p*_*j *_is the frequency of the *j*^th ^pattern [28]. PIC was used as a tool to measure the information that a given marker locus could provide for the pool of genotypes, while DP was used as a quantification tool to measure the efficiency of a given marker for the discrimination of genotypes; i.e., the probability that two randomly chosen individuals have different patterns.

#### Genetic similarity analysis

To evaluate the genetic similarity among all the genotypes, the polymorphic bands were used to construct a binary matrix. The EST-SSR-based genetic similarity (SSR-GS) among all of the genotypes was estimated according to the Jaccard's similarity coefficient [29]. The corresponding genetic similarity matrix was used to generate a dendrogram based on the Unweighted Pair Group Method with the Arithmetic Average (UPGMA) algorithm, as suggested by Sneath and Sokal [30]. All analyses were carried out using NTSYSpc 2.11X [31]. A bootstrap analysis with 10,000 random samplings was applied to estimate the reliability of the dendrogram branches using BOOD version 3.0 [32].

## Results and Discussion

### Development of potentially functional SSR markers

According to the search criteria adopted, a total of 2005 clusters containing SSRs were found in the SUCEST database. These sequences were considered useful because they contained dinucleotide, trinucleotide or tetranucleotide repeats as described by literature [18]. Oligonucleotide primer pairs were successfully synthesized and tested for 365 of the ESTs that contained trinucleotide motifs. The sequences were selected based on the size of the individual repeat units and the expected homology to known genes. The EST-SSRs were initially tested in agarose gels to verify the presence and quality of the product amplification of a predicted size. Afterwards, the selected EST-SSRs were screened in 6% denaturing polyacrylamide gels to identify a high-quality banding pattern. The details regarding the primer pair sequences and the product size of the SSR motifs for the selected markers are described in Additional file 1.

The used molecular markers derived from EST is a strategy that allows a direct mapping interest genes [19,20,33-35] and the discovery of the functional portion of the genome [21]. In this study, a large number of sugarcane ESTs derived from the SUCEST database were exploited to generate a set of functionally associated markers for sugarcane. Expressed sequences from sugarcane were used to develop EST-SSR markers that are expected to improve the detection of marker-trait associations because they are part of the transcribed domain of the genome. In recent years, emphasis has been placed on the development of functional molecular markers in order to assess variation in wild relatives or to establish genetic similarities between genotypes for breeding purposes, it is valuable to have information regarding the variability of specific gene that potentially affect important breeding traits.

### Functional characterization EST-SSR

Of the 197 ESTs sequences compared to sequences from GenBank using the BLASTX algorithm, 65.98% had homology with a known protein and 23.62% had homology with a putative protein. The remaining 10.40% of the sequences did not possess homology with any sequence in the database.

The unigenes identified in this study had significant similarity with genes from other plant species. The majority of the similarities were found to *Sorghum bicolor, Oryza sativa*, and *Zea mays*. The enrichment of similarities with genomes these species is likely because they have sequenced genomes, a large number of expressed sequences deposited in databases, and a close phylogenetic relationship with sugarcane. Data regarding the homologies to each of the EST-SSR markers are shown in Additional file 2.

**Figure 1:**
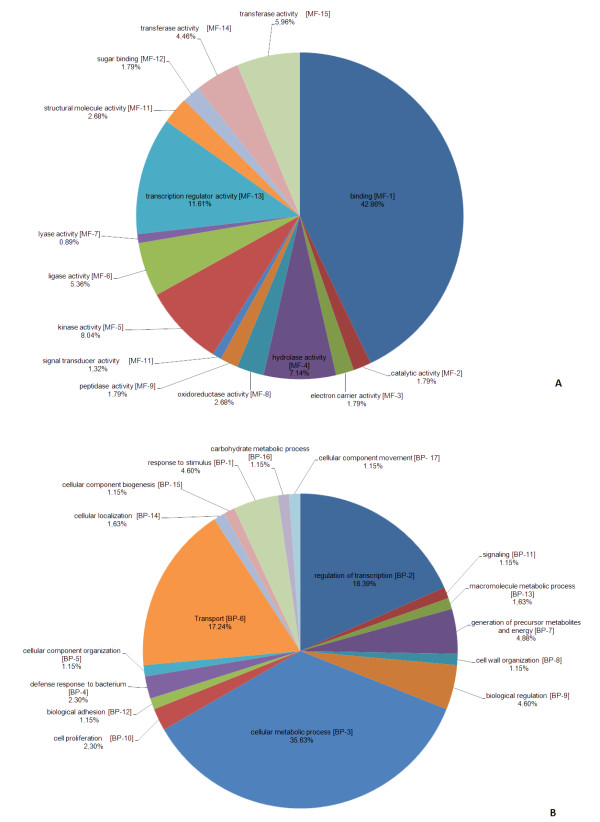
**Ontology categories of sugarcane ESTs**. ESTs were analyzed using the AmiGO browser in the Gene Ontology (GO) database. The categories shown are molecular function (A) and Biological Processes (B).

Ontology has been used in many model organisms to annotate the function of genes and their products and may be represented in terms of Molecular Function (MF), Biological Process (BP) and Cellular Component (CC). We made notes of the MF and BP for each EST. Based on the 130 ESTs that had homology with a known protein, 97 (74.61%) of these ESTs possessed a known molecular function and/or biological process. Of these, 78 (59.54%) ESTs possessed a known biological process and 88 (67.18%) ESTs possessed a known molecular function. The molecular functions most occurring were characterized into the following groups: binding (42.86%), transcription factor activity (11.61%) and kinase activity (8.04%). Regarding the biological processes, the ESTs characterized in this work are mainly involved in cellular metabolic process (35.63%), regulation of transcription (18.39%) and transport (17.24%). The ontology category, based on the molecular function and biological processes of EST-SSR markers, is represented graphically in Figure 1. The functional annotation (MF and BP) for each EST-SSR marker is listed in additional file 2.

We identified several EST-SSR markers that can be associated with genes that play important physiological roles in plants. Some of the clusters that gave rise to these markers shared homology with were related to proteins deposited in GenBank. For example, the SCB207 marker is derived from an EST with homology to a gene (ERECTA) that regulates the efficiency of sweating. This gene coordinates sweating and photosynthesis [36] and indirectly influences productivity. The SCB218 marker is associated with the acid soluble invertase protein, an enzyme that limits the accumulation of sugars such as sucrose in the immature internodes of sugarcane stalks [37]. We also identified SCB324 and SCB336 as markers associated with the MoS2 protein. MoS2 was identified in *Arabdopsis thaliana *and is essential in the innate immune response, especially at the beginning of the cascade that is activated in response to pathogens [38]. Finally, the SCB487 marker may be associated with a gene for sugar transport.

### Polymorphism revealed by EST-SSR

A total of 365 primer pairs flanking SSR regions were designed to detect polymorphisms across the set of 18 sugarcane genotypes (15 genotypes came from a interspecific hybrid *Saccharum spp*., and the remaining from 3 different species: *S. officinarum, S. barberi*, and *S. sinense *). In 287 sequences (78.6%) of the total developed, clear, strong DNA fragments were produced by PCR amplification. The PCR reactions using the remaining 78 (21.4%) primer pairs failed, resulting in weak DNA amplification or an unexpected fragment size. Of the 287 selected primer pairs that produced a clear product, 197 (68.6%) were polymorphic and 90 (31.4%) were monomorphic. The EST-SSR amplifications revealed high levels of polymorphism and detected 1261 alleles with a range of 2 alleles (SCB168, SCB180, SCB187, SCB192, SCB193, SCB195, SCB204, SCB210, SCB231, SCB232, SCB234, SCB240, SCB241, SCB250, SCB293, SCB302, SCB306, SCB345, SCB377, SCB377, SCB379, SCB402, SCB405, SCB407, SCB416, SCB418, SCB445, SCB449 and SCB459) to 18 alleles (SCB344), the mean was 6.4 alleles per EST-SSR (Additional file 1).

The PIC values ranged from 0.21 (SCB345) to 0.92 (SCB344), and the average PIC was 0.69. The DP was high for the majority of the EST-SSRs, with an average value of 0.80. A maximum value was found for eight EST-SSRs (SCB181, SCB246, SCB285, SCB330, SCB334, SCB373, SCB374 and SCB466) (Additional file 1).

Similar PIC values were reported by Oliveira et al. [21] using the same set of sugarcane varieties ranged from 0.16 to 0.94 with an average 0.73. While Cordeiro et al. [39], considering sugarcane Hybrids, *S. officinarum *and *S. sinense*, found PIC values 0.77, 0.68 and 0.70 respectively, suggesting that most of these markers will be useful in the assessment of the genetic diversity in sugarcane because of their high potential for discrimination and genetic mapping.

The amplification success rate of the EST-SSRs was 78.6% and is similar to those rates reported in previous studies with sugarcane (60%, [25]; 77%, [18]; 62%, [19]; 70%, [21]), barley (64%, [14]), wheat (70%, [40]), white clover (71%, [41]) and coffee (80.9%, [42]). The remaining (21.4%) primer pairs failed to amplify DNA or resulted in weak DNA amplification. The amplification of a larger fragment than expected could reflect the presence of introns within the genomic DNA sequence. The lack of amplification could reflect the presence of long introns between the sequences homologous to the primers in the genomic DNA. Other reasons for a failed amplification include the absence of the allele or the divergence of the sequence in the SSR flanking region.

The high values obtained are typical of SSR markers, and these SSRs may be capable of detecting larger variation as reported in sugarcane gSSRs [19,43] and sugarcane EST-SSRs [18,21]. Most of the markers isolated will be useful in determining sugarcane parentage and making clonal assessments; the high levels of polymorphism indicate that they will most likely segregate in crosses, giving them great potential in use for discrimination and for the construction of genetic linkage maps.

### Genetic similarity using EST-SSR

**Figure 2:**
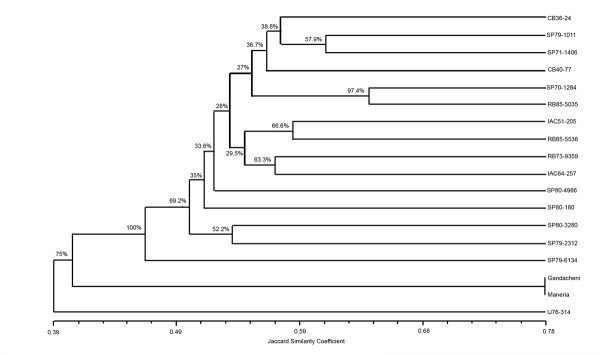
**Dendogram representing the relationship between the 18 genotypes of sugarcane, using Jaccard`s similarity coefficients**.

To test the potential of the EST-SSRs to act as polymorphic markers in genetic studies, allelic data from the 197 EST-SSRs were used to construct a dendrogram (Figure 2) with the studied sugarcane varieties. Genetic similarities based on the Jaccard coefficient were calculated with 1023 polymorphic markers amplified by the EST-SSRs, and they varied from 0.39 to 0.78. The data generated by these markers were not sufficient to differentiate between the two species *Gandacheni *(*S. barberi) *and *Maneria *(*S. sinense) *(0.78), but they could differentiate these two species from *S. officinarum*. The dendrogram shown in this work is in accordance with the origin of the analyzed sugarcane varieties because *S. sinense *and *S. barberi *are usually considered natural hybrids between *S. spontaneum *and *S. officinarum *[44]. In general, the clustering of the analyzed genotypes is in agreement with previous works [18,19,21], validating the new EST-SSRs. The novel EST-SSR markers developed in the present work represent a suitable genetic resource for assessing sugarcane parentage, clonal evaluation, genetic analysis, and to improve direct estimates of functional diversity.

By analyzing the dendogram, observed that most genotypes are grouped because sugarcane species share a narrow genetic base. Commercially cultivated sugarcane is an interspecific hybrid between *S. officinarum *and *S. spontaneum*. About 80% of the genome is contributed by S. *officinarum*, 10% - 15% is contributed by S. *spontaneum *and only 5% - 10% is thought to consist of recombinant chromosomes [45,46]. This may explain why the IJ76-314, that is a *S*. *officinarum*, cannot be placed in any group and why *Gandacheni *and *Maneria*, both *S. officinarum*, cannot be distinguished themselves, remaining in the same group.

These results corroborate the observed by Lima et al. [22], which observed a tendency for the cultivars to group together with others obtained from the same cross. For example, the *NA56-79 *cultivar is associated with crossings that have generated the cultivars *SP79-1011 *and *SP71-1406 *(Table 1); these cultivars clustered with a similarity coefficient of around 0.60 (Figure 2). Another example includes the genotypes *SP70-1284 *and *RB85-5035*. The former is the parent of the cross that generated the latter (Table 1), and they clustered with a similarity coefficient of around 0.65 (Figure 2).

## Competing interests

The authors declare that they have no competing interests.

## Authors' contributions

TGM, EAC, MCM and CBCS carried out the molecular genetic studies, participated in the sequence alignment, performed the statistical analysis and drafted the manuscript. HRCAN, KMO and LRP carried out the molecular genetic studies. MM performed the statistical analysis. AAFG and APS conceived of the study, and participated in its design and coordination and helped to draft the manuscript. All authors read and approved the final manuscript.

## Supplementary Material

Additional file 1 **Description of EST-SSR primer pairs characterized in sugarcane**. The table presents the markers characterized in sugarcane, as primer sequences, annealing temperatures, number of alleles, expected size, allele range, Polymorphism content and power discrimination. Click here for file

Additional file 2 **Homology and annotation functional EST-SSRs markers**. The functional markers were analyzed for their similarity to proteins deposited in GENBANK, further their molecular function and biological process. Click here for file
